# CCAT 1- A Pivotal Oncogenic Long Non-Coding RNA in Colorectal Cancer

**DOI:** 10.3389/bjbs.2023.11103

**Published:** 2023-03-21

**Authors:** Xiew Leng Liau, Shamala Salvamani, Baskaran Gunasekaran, Dinesh Kumar Chellappan, Anthony Rhodes, Vaidehi Ulaganathan, Yee Lian Tiong

**Affiliations:** ^1^ Division of Applied Biomedical Sciences and Biotechnology, School of Health Sciences, International Medical University, Kuala Lumpur, Malaysia; ^2^ Department of Biotechnology, Faculty of Applied Sciences, UCSI University, Kuala Lumpur, Malaysia; ^3^ Department of Life Sciences, School of Pharmacy, International Medical University, Kuala Lumpur, Malaysia; ^4^ Department of Pathology, Faculty of Medicine, University Malaya, Kuala Lumpur, Malaysia

**Keywords:** biomarker, long non-coding RNA, colorectal cancer, CCAT 1, c-MYC

## Abstract

Colorectal cancer (CRC) is ranked as the third most common cancer and second deadliest cancer in both men and women in the world. Currently, the cure rate and 5-year survival rate of CRC patients remain relatively low. Therefore, discovering a novel molecular biomarker that can be used to improve CRC screening, diagnosis, prognosis, and treatment would be beneficial. Long non-coding RNA colon cancer-associated transcript 1 (CCAT 1) has been found overexpressed in CRC and is associated with CRC tumorigenesis and treatment outcome. CCAT 1 has a high degree of specificity and sensitivity, it is readily detected in CRC tissues and is significantly overexpressed in both premalignant and malignant CRC tissues. Besides, CCAT 1 is associated with clinical manifestation and advanced features of CRC, such as lymph node metastasis, high tumor node metastasis stage, differentiation, invasion, and distant metastasis. In addition, they can upregulate oncogenic c-MYC and negatively modulate microRNAs *via* different mechanisms of action. Furthermore, dysregulated CCAT 1 also enhances the chemoresistance in CRC cells while downregulation of them reverses the malignant phenotypes of cancer cells. In brief, CCAT 1 serves as a potential screening, diagnostic and prognostic biomarker in CRC, it also serves as a potential therapeutic marker to treat CRC patients.

## Introduction

Colorectal cancer (CRC) is ranked as the third most common cancer and second deadliest cancer in both men and women in the world. In 2020, approximately 1,931,590 people are diagnosed with CRC and 935,173 people died because of CRC worldwide. In Malaysia, CRC is also the third-leading cancer and is recorded to have 6,597 new cases and cause 3,462 deaths in 2020 (1).

CRC is usually caused by a gradual buildup of gene mutations or sometimes by changes in epigenetic processes, which leads to abnormal activation of oncogenes and inactivation of tumor suppressor genes (2) It begins from benign adenomatous polyps to advanced adenoma with high-grade dysplasia or carcinoma *in situ*, invasive adenocarcinoma and ultimately, metastasizes to distant organs such as the liver (3).

Although the treatment of CRC has been improved, the cure rate and 5-year survival rate for CRC patients are still relatively low as many of them have already been diagnosed with stage III or IV CRC at their first visit (4). In addition, after potentially curative surgery or adjuvant therapies, one-third of the patients will still relapse (5). Therefore, early detection of benign colon lesions and recurrence of disease through an effective screening method, such as using molecular biomarkers, is important to increase the chance of survival and significantly improve the overall outcome in CRC patients (6).

To date, a lot of evidence has revealed that long non-coding RNAs (lncRNAs) molecules are aberrantly expressed in CRC tissues or cells (7). LncRNAs are a class of regulatory non-coding RNAs (ncRNAs) that have the highest diversity, they are at least 200 nucleotides long and possess high tissue specificity as they are regulated by specific regulatory systems that are different from protein coding genes (8). As the name suggests, lncRNAs do not encode protein as they lack functional open reading frames (ORFs) (9). However, they can act as regulatory molecules to modulate cellular or biological processes such as cell proliferation, differentiation, and apoptosis through interacting with other cellular macromolecules like RNA, DNA, and proteins, and through regulating gene expression epigenetically, transcriptionally, and post-transcriptionally (10). LncRNAs are widely dysregulated in cancer, their expression level in cancer depends on whether they act as tumor driving genes or tumor suppressor genes, and dysregulated lncRNA triggers tumor carcinogenesis including CRC (11).

In 2012, Nissan et al. discovered a new oncogenic lncRNA that is aberrantly overexpressed in colon cancer using Representational Difference Analysis (RDA), cDNA cloning, and rapid amplification of cDNA ends (RACE) (12). This lncRNA is named colon cancer-associated transcript-1 (CCAT 1) or LOC100507056 and it is 2,628 nucleotides long (12). Since then, many studies also revealed that CCAT 1 is overexpressed in other human cancers, such as gastric cancer (13), lung cancer (14), breast cancer (15), ovarian cancer (16), gall bladder cancer (17), hepatocellular carcinoma (18), prostate cancer (19) and acute myeloid leukemia (20). Other than human cancers, CCAT 1 is also significantly expressed in inflammatory bowel diseases such as ulcerative colitis (UC) and Crohn’s disease (CD) (21). In CRC, dysregulated CCAT 1 has been discovered to promote tumorigenesis by facilitating proliferation, metastasis, and anti-apoptosis of CRC cells through multiple mechanisms (12). Moreover, dysregulation of CCAT 1 also affects the developing chemoresistance in CRC cells (22). Therefore, it is suggested that CCAT 1 may be a potential molecular biomarker in screening, diagnosis, prognosis and act as a target for CRC treatment.

In this review, we describe the characteristics and identifications of CCAT 1, and its potential role in screening, diagnosis, prognosis and treatment of CRC. The mechanism of actions of CCAT 1 and the factors that cause CCAT 1 dysregulation in CRC are also elucidated. In the last, we describe the biological functions of CCAT 1 in CRC tumorigenesis, and how CCAT 1 contributes to the chemoresistance of CRC cells.

## CCAT 1

CCAT 1 is an oncogenic lncRNA, its gene is located on human chromosome 8q24 (chr.8q24) region, specifically 8q24.21 nearby the *c-MYC* gene, which is one of the well-studied oncogenes (23). CCAT 1 RNA contains three short ORFs, which are nucleotides 95–208; 310–519 and 1,621–1,770 respectively. However, none of them can encode protein (12). In addition, CCAT 1 is multiexonic, it contains two exons which are nucleotides 1–288 and 289–2,612 respectively and is capped at the 5′ end and polyadenylated at the 3′ end (12, 24). Moreover, the promoter region of CCAT 1 contains an evolutionarily conserved enhancer box (E-box) (25). This E-box can be bound by c-MYC transcription factor which plays an extensive role in the initiation and development of most cancers (25). Furthermore, CCAT 1 consists of a short sequence at the 3′ end called microRNA response element (MRE) that complements the seed region or 5′ portion of certain micro-RNAs (miRNAs). It regulates the biological function of those miRNAs by targeting and interacting with them (26, 27) Taken together, all the characteristics stated above allow CCAT 1 to play roles in CRC tumorigenesis.

### Isoforms: CCAT1-S and CCAT1-L

According to the GENBANK nucleotide sequence database, CCAT 1 produces two isoforms: short isoform CCAT1-S and long isoform CCAT1-L (28). CCAT1-S is 2,628 nucleotides long, it was identified in colon cancer in 2012; whereas CCAT1-L is 5,200 nucleotides long, it was discovered in CRC in 2014 (12, 24). The short isoform CCAT1-S is also referred as CCAT 1 or cancer-associated region long noncoding RNA-5 (CARLo-5), it is named as CCAT1-S after the long isoform CCAT1-L is discovered (12). Same as CCAT1-S, CCAT1-L is also transcribed from 8q24, 515 kb upstream of *c-MYC* gene (MYC-515), a tumor type-specific super enhancer region of *c-MYC* with a length of 150 kb (24). Therefore, CCAT1-L may also be identified as an enhancer-derived RNA (eRNAs) for *c-MYC* (24). In addition, CCAT1-L also contains two exons, and it is 3′-capped and 5′-polyadenylated although eRNAs are not spliced or polyadenylated in general (28).

The relationship between CCAT1-S and CCAT1-L and their characteristics were further studied by Xiang et al. They found out that there is a spatial structure overlap between CCAT1-L and CCAT1-S in which two exons of CCAT1-L overlapped with CCAT1-S, and reduced CCAT1-L causes an immediate disruption of CCAT1-S (24) Therefore, CCAT1-S is suggested to be derived from CCAT1-L and there may be a correlation between them. In addition, both are localized in different subcellular compartments. After transcription, CCAT1-S is transferred to the cytoplasm whereas CCAT1-L is retained in the nucleus, more accurately, near or at its site of transcription (24). Moreover, CCAT1-S is highly expressed in CRC (12), gastric cancer (13), gallbladder cancer (17), and hepatocellular cancer (18) whereas CCAT1-L is only overexpressed in CRC (24). Recent evidence reveals that CCAT1-L is also overexpressed in gastric adenocarcinoma and hepatocellular carcinoma, hence CCAT1-L is no longer specifically expressed only in CRC (29, 30).

### Potential Roles of CCAT 1 in CRC

CCAT 1 is found significantly overexpressed in both early and late stages of CRC patients (31, 32). There was a significant differential expression of CCAT 1 in CRC tumour tissues and normal adjacent tissues in which CCAT 1 is upregulated in CRC tumour tissues as compared to normal colon mucosa (32). Current studies in Singapore showed that the expression of CCAT 1 in patients’ tumours was hundreds of times higher as compared to their matched normal mucosa (33). Besides, high expression of CCAT 1 can be detected in every stage of the mucosal adenoma-carcinoma sequence in CRC, either pre-malignant or malignant tissues, such as benign adenomatous polyps, primary colon adenocarcinoma including lymph nodes and liver metastases (31). In addition to colorectal tumor tissues, CCAT 1 is also highly expressed in peripheral blood mononuclear cells (PBMC) of colon cancer patients (12). Furthermore, the high expression level of CCAT 1 can also be detected in colon cancer-associated lymph nodes. Siddique et al. also revealed that the plasma CCAT 1 expression in CRC patients exceeds 4.54-fold than in normal individuals (34). Not only in tissue and plasma samples, the expression of CCAT 1 also showed significant differences in stool samples from CRC patients and healthy individuals, with the former being 4.5 times higher than the latter (35). The extremely high ratio of tumour to normal tissue highlights that CCAT 1 is specific and these findings suggest that CCAT 1 may be used as a screening and diagnostic biomarker for CRC tissues. Intriguingly, Zhao et al. revealed that using CCAT 1 together with another lncRNA HOTAIR can improve CRC screening and detect CRC at an early stage as possible. The authors agree with the high diagnostic power of plasma CCAT 1, with the indication of 85.3% specificity and 75.7% sensitivity (36). Besides, Kam et al. demonstrate that thiazole orange-peptide nucleic acid molecular beacon (TO-PNA-MB) complementary to CCAT 1 detects CRC in human biopsies based on the FISH method and the results showed satisfactory results in which higher fluorescence intensity was seen in benign adenoma and adenocarcinoma tissues as compared to their matched normal colonic tissues (37).

Secondly, although the utility of prognostic biomarkers in a clinical setting is less than screening and diagnostic biomarkers, they are nevertheless useful in evaluating a patient’s likely outcome regardless of treatment (38). A meta-analysis demonstrated that CCAT 1 expression affects CRC patients’ clinical stage and their overall survival (OS) (39). Patients that have high CCAT 1 expressed in CRC tissues have shorter survival times and poorer disease-free survival (40). In addition, increased CCAT 1 is also significantly correlated with advanced clinical features of CRC, such as lymph node metastasis (LNM), high tumor node metastasis (TNM) stage, differentiation, microvascular invasion, and distant metastasis (41). In short, CCAT 1 can be utilized as a potential prognostic biomarker to evaluate patients’ clinical outcomes and predict their survival rate regardless of the metastasis stage and treatment in CRC.

Strikingly, CCAT 1 can also be used to predict the therapeutic effects of CRC patients so that a suitable and effective treatment can be proposed. Specific targeted treatment can be restricted to CRC patients expressing CCAT 1 (38). JQ-1 treatment is a targeted therapy that uses bromodomain and extra-terminal (BET) protein inhibitors to target BET proteins (42). During the development of CRC, BET protein accumulates and binds at super-enhancers of *c-MYC*, thereby activating the transcription of *c-MYC* gene in a tumor type-specific and lineage-dependent manner (43). Since CCAT 1 is situated at 500 kb upstream of *c-MYC* promoter, which is the super-enhancer region of *c-MYC*, it may be bound by this BET protein family (43). CCAT 1 is significantly downregulated in CpG island methylator phenotype positive (CIMP+) colon cancer cells upon JQ1 treatment, this indicates that CCAT 1 is sensitive to BET inhibitors and can be directly regulated by BET protein (43). Since CCAT 1 is sensitive to BET inhibition and its expression predicts JQ1 sensitivity as well as BET-mediated *c-MYC* regulation, it can serve as a potential biomarker in which its expression level can help identify patients who can well-respond and are most likely to benefit from BET inhibitor treatment (43). Undeniably, this patient selection strategy will be very useful in clinical trials. However, as super enhancers cannot be detected consistently in human tissues, CCAT 1 cannot be used as a predictive biomarker for companion diagnostic assay (43).

In addition to screening, diagnosis and prognosis, CCAT 1 may serve as a target for onco-lncRNA targeted therapy, which could be a promising treatment option for CRC patients (44). Study shows that the expression of CCAT 1 can be significantly reduced by siRNA. Downregulation of CCAT 1 upregulates the expression of cyclin dependent kinase inhibitor 1A (CDKN1A) mRNA, which regulates G1 cell cycle arrest and leads to a reduction in colon cancer cell proliferation (14, 45)Moreover, by knocking down CCAT 1, the malignant characteristic of CRC cells, such as migration and invasion can be reversed (28). For instance, the authors discovered that Ginsenoside Rg3, an anti-cancer compound, can downregulate CCAT 1 thereby inactivating the phosphatidylinositol 3-kinase/protein kinase B (PI3K/AKT) pathway and eventually inhibiting proliferation, migration and invasion of CRC cells (46). Furthermore, downregulation of CCAT 1 also induces CRC cell apoptosis by increasing proapoptotic protein Bcl-2-associated X protein (BAX) expression levels *via* p53 signaling pathway (47).

In brief, CCAT 1 may serve as a useful biomarker in screening, diagnosis and prognosis of CRC; it may also serve as a target for onco-lncRNA targeted therapy for CRC patients and facilitate in selecting patients that can respond well to a specific treatment. However, targeting or using CCAT 1 as a treatment approach requires a more comprehensive knowledge of its mechanisms of action and its biological functions in CRC, and they are described below.

## Mechanisms of Action of CCAT 1 in CRC

lncRNAs are important gene expression regulators. They function as protein scaffolds, transcription coactivators or inhibitors, and mRNA decoys or microRNA sponges to regulate their expression thereby modulating biological or cellular processes (48). Considering that CCAT 1 is a lncRNA, it should also exhibit some of these functions. Until now, there are four mechanisms of action of CCAT 1 found in cancer, however, only two mechanisms of action of CCAT 1 are known in CRC (23). As mentioned, CCAT1-L and CCAT1-S are localized in different subcellular compartments, and this leads them to exert different regulatory roles at their particular sites of action.

**FIGURE 1:**
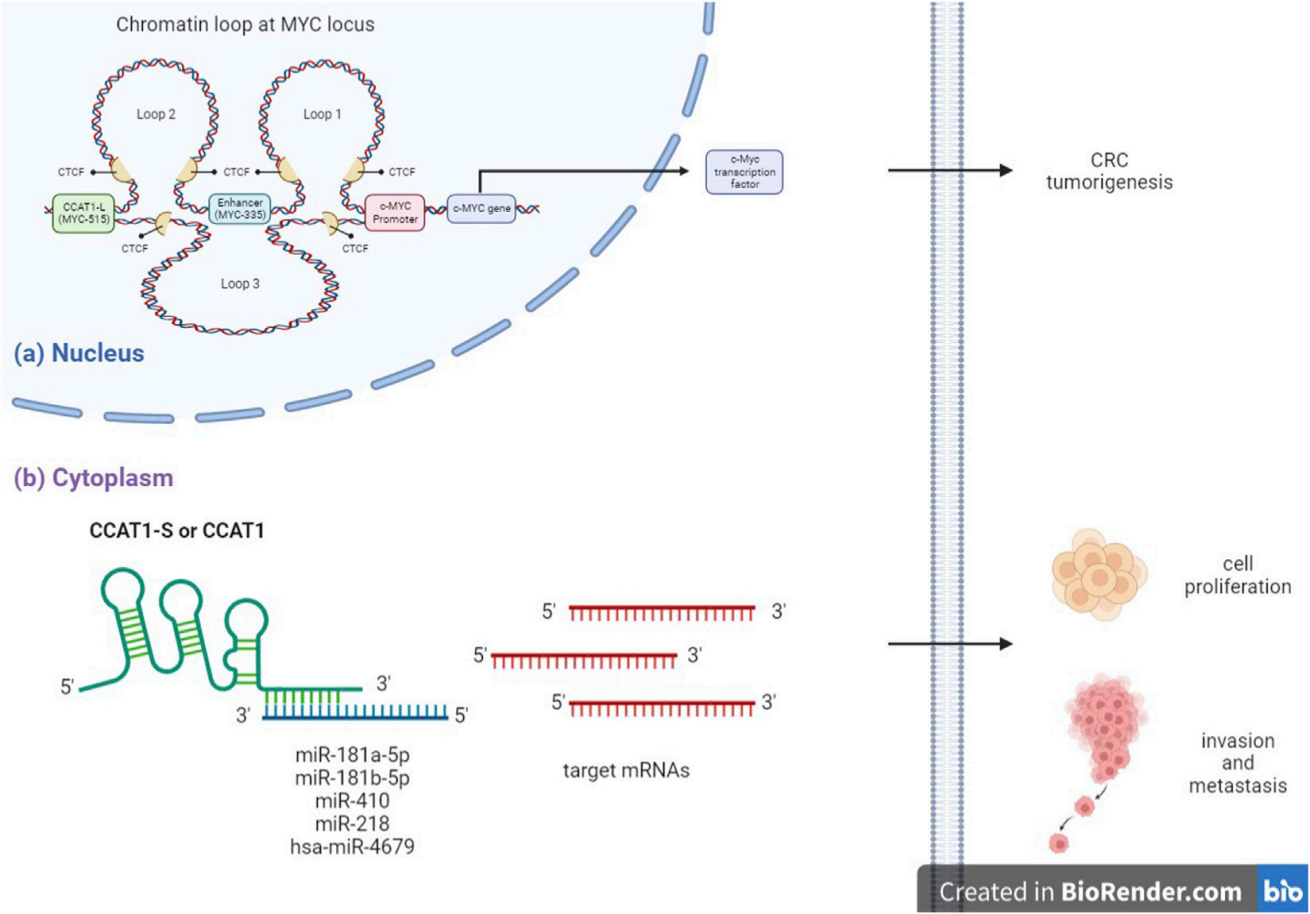
Mechanism of action of CCAT 1 in CRC. **(A)** Nuclear lncRNA CCAT1-L, transcribed from distal tumor type-specific super-enhancer of c-MYC (MYC-515), interacts with CCCTC-binding transcription factor (CTCF) subsequently mediating intra-chromosomal interaction of well-studied oncoproteins c-MYC, which plays important role in CRC tumorigenesis. **(B)** Cytoplasmic lncRNA CCAT1-S or CCAT1 directly interacts with tumor suppressive miRNAs subsequently regulating miRNA target mRNAs translation and eventually promoting colon cancer cell proliferation, invasion and metastasis.

Firstly, nuclear lncRNA CCAT1-L acts as an oncogene by binding to CCCTC-binding transcription factor (CTCF), regulating the intra-chromosomal interaction of well-studied oncoproteins c-MYC causing CRC tumorigenesis. Whereas cytoplasmic lncRNA CCAT1-S or CCAT 1 act as an oncogene by directly interacting with tumor suppressive miRNAs thereby upregulating miRNA target mRNAs translation and eventually promoting CRC proliferation, invasion and metastasis (Figure 1) (24, 49).

### CCAT1-L Forms Long Range Chromatin Loop With the c-MYC Oncogene at the 8q24 Locus

Remarkably, there are several loci commonly found mutated in cancer, including the 8q24 locus (50). Within chromosome 8q24, there is a segment termed “gene desert” with approximately 3Mb long (51). This segment encompasses single nucleotide polymorphisms (SNPs) that cause increased susceptibility to various cancers, such as CRC, prostate cancer, breast cancer, esophagus cancer, ovarian cancer and pancreas cancer (52). Intriguingly, *c-MYC* gene is located just a few hundred kilobases telomeric to those mutational hot spots within this chromosome. It plays a vital role in maintaining CRC cell identity and promoting oncogenic transcription (51, 53). *c-MYC* upstream regulatory elements such as enhancers and super-enhancers that are located at MYC-515 regulate the transcription of *c-MYC* gene in a tissue or tumor type-specific manner (54). This super-enhancer involves maintaining the stability of chromatin looping at *MYC* locus through the formation of two chromatin loops with *c-MYC* promoter and a transcriptional enhancer for *c-MYC* gene (MYC-335) (24).

Given that protein coding genes are rarely found in gene desert near *c-MYC*, coupled with the emergence of lncRNAs, many researchers have attempted to discover lncRNAs that may regulate c-MYC to promote CRC tumorigenesis (54). To date, multiple lncRNAs that regulate *c-MYC* gene expressions such as CCAT 1, CCAT1-L and Colon Cancer Associated Transcript 2 (CCAT2) have been discovered (55). Interestingly, studies have shown that c-MYC expression in the tumour was significantly correlated to CCAT 1 (33)*.* As mentioned before, CCAT1-L is transcribed from MYC-515, therefore, this allows it to play a role in regulating local gene expression and organizing chromatin structure (24). Furthermore, CCAT1-L is observed accumulating in-cis at or near its site of transcription in the nucleus (24). It can act as a cis-regulatory element to transcriptionally activate *c-MYC* by binding to it. Taken together, CCAT1-L serves as an enhancer-derived RNA (eRNA) and chromatin regulator for *c-MYC* by upregulating *c-MYC* transcription and promoting long range chromosomal interactions at *MYC* locus (28).

#### Mechanism of Action of c-MYC

*c-MYC* is a tumor driving gene that was found overexpressed in numerous cancers including CRC. It is located at ∼335kb telomeric of rs6983267 on chromosome 8q24, single nucleotide polymorphisms (SNPs) that were found to be associated with CRC (56, 57). Its expression is regulated by *c-MYC* proto-oncogene, which gives an immediate response after the activation of numerous ligand-membrane receptor complexes as its location is at the intersections of many growth-promoting signal transduction pathways (54, 56). *c-MYC* oncogene is a downstream regulatory gene of the PI3K/Akt signaling pathway, which is one of the most important pathways in CRC, and its activation reduces cell apoptosis and promotes cell proliferation (58, 59). *c-MYC* oncogene encodes an oncoprotein called c-MYC, which is a general transcription factor that regulates the expression of the gene that changes the characteristics of epithelial stem cells of colon tissues (60). As a typical transcription factor, c-MYC oncoprotein binds to promoters of genes or recruits histone acetyltransferases (HATs) epigenetically to regulate gene expression (54). c-MYC regulates 15% of all genes by binding to the enhancer box (E-box) with the sequence of 5′-CACGTG-3′ which is present in the promoter region of those genes, and this includes CCAT 1 (61). To function, c-MYC dimerizes with a protein called Max to form a transcriptional competent factor complex. This complex then binds target DNA sequences or E-boxes of target genes which are involved in cell proliferation and growth, differentiation, apoptosis and adhesion such as *cyclin-dependent kinase 4 (CDK 4)* (55). As such, c-MYC augments their expression thereby promoting cell proliferation and growth.

Under normal conditions, *c-MYC* gene expression is strictly controlled by many transcriptional regulatory motifs that are found within its promoter region with different mechanisms of action (54). When chromosomal translocations and aberrant signal transduction occur, *c-MYC* transcription is dysregulated, resulting in sustained *c-MYC* expression and an increased level of c-MYC transcription factor (62). Then, the increased c-MYC binds to E-boxes of target genes to command them, enabling the cells to grow and divide persistently, thus initiating the neoplasia formation (54).

#### How CCAT 1-L Regulates *c-MYC* Transcription

So how does CCAT1-L regulate *c-MYC* transcription when it needs to across 515 kilobases, which is such a large genomic distance? The answer will be the formation of long-range chromatin loops, which have been recognized to bring genes side by side to the enhancers (63). For that reason, CCAT1-L positively regulates the expression of *MYC* transcription by forming and promoting long-range chromatin interactions between *MYC* and its upstream regulatory elements (24). Xiang et al. demonstrated that CCAT1-L participates in the first two of the three chromatin loops generated at *MYC* locus: loop 1 connects *c-MYC* promoter to MYC-335; loop 2 connects MYC-335 to MYC-515, loop 3 connects MYC-515 to *c-MYC* promoter (24). CCAT1-L is shown to play a role in maintaining the stability of enhancer-promoter looping at *MYC* locus in CRC cancer cells by recruiting a chromatin loop forming factor called CCCTC-binding factor (CTCF) (23). When CCAT1-L interacts with CTCF, it promotes the chromatin interactions between the *c-MYC* promoter and its upstream enhancers, thereby activating the transcription of *c-MYC*. Moreover, studies reveal that in-cis accumulation of this lncRNA further promotes *c-MYC* transcription and enhances CRC tumorigenesis (24). In contrast, knockdown of CCAT1-L decreases *c-MYC* expression. When CCAT1-L is knocked down, it reduces the chromatin interactions between *c-MYC* promoter and its enhancers, therefore, *c-MYC* transcription will be reduced (24). Then, reduced expression of *c-MYC* leads to reduced translation of c-MYC, which eventually causes a reduction in CRC cell proliferation.

### CCAT 1 Functions as a Molecular Sponge for miRNAs

A growing number of reports suggest that both lncRNAs and miRNAs have participated in CRC development and progression through lncRNA-miRNA-mRNA cross talk. They act as competing endogenous RNAs (ceRNAs) to regulate CRC cell proliferation, differentiation and apoptosis (64,65). miRNAs are small and highly conserved non-coding RNAs with a length of 18–24 nucleotides that regulate the translation and stability of specific target mRNAs (66). They can either be tumor suppressor genes or oncogenic miRNA, depending on the microenvironment in the cells that they are expressed (66). Recent studies suggest that the relationship between CCAT 1 and miRNAs in CRC is double negative feedback or reciprocal repression (18). Cytoplasmic lncRNA CCAT 1 functions as a molecular sponge or decoy for miRNAs. It changes the biological function of miRNA at the transcriptional level, thereby changing the expression of miRNA target genes indirectly (18). CCAT 1 contains a binding site for miRNAs which is the miRNA response element (MRE) at the 3′ end. It acts as a miRNA sponge that bind to certain miRNAs to inhibit their endogenous suppressive or oncogenic effects on their targets (49). As a result, CCAT 1 indirectly increases the expression of miRNA target genes (18).

Remarkably in CRC, several miRNAs are found to be the potential targets of CCAT 1, such as miR-124, miR-490-3p, miR-194, miR-24 and miR-181a-5p (47). However, only miR-181a-5p has inversely correlated with CCAT 1 expression (47). Subsequently, other studies reveal that miR-181b-5p, miR-218, miR-410 and hsa-miR-4679 are also the functional targets of CCAT 1. By binding and sponging these miRNAs, CCAT 1 induces the proliferation, invasion, and metastasis of CRC cells. It also inhibits cell cycle arrest and apoptosis of CRC cells (Table 1) (47, 49, 67–69).

**TABLE 1:** miRNAs in CRC that are functional targets of CCAT 1

miRNA	Types of miRNA	Expression in CRC	Experimentally validated microRNA targets	Functions	Ref.
miR-181a-5p	Tumor suppressor	Low	P53	Suppress cell proliferation, mobility and invasion, and promote cell apoptosis	(47)
miR-181b-5p	Tumor suppressor	Low	Tumor suppressor candidate 3 (TUSC3)	Suppress cell proliferation, migration and invasion	(49)
miR-218	Tumor suppressor	Low	Vascular endothelial growth factor (VEGF)	Inhibit cell viability, promote apoptosis and reduce VEGF expression	(67)
miR-410	Tumor suppressor	Low	Inositol-Trisphosphate 3-Kinase B (ITPKB)	Suppress cell proliferation, migration and invasion, promote apoptosis	(68)
hsa-miR-4679	Tumor suppressor	Low	Guanine nucleotide-binding protein, gamma 10 (GNG10)	Suppress cell proliferation, migration and invasion, promote apoptosis	(69)

## Factors Involved in Dysregulation of CCAT 1 in CRC

Although many studies have revealed that CCAT 1 is associated with CRC tumorigenesis, the underlying mechanism that causes CCAT 1 dysregulation in CRC has not been dealt with in depth. Many lines of evidence suggest that aberrant expression of lncRNAs can be caused by genetic alterations as well as epigenetic regulation. Indeed, SNPs, copy number alterations, or mutations within the non-coding genome alter the transcription of lncRNA (10, 70). Several SNPs are related to the expression of cancer-associated lncRNAs including CCAT2 and Prostate cancer-associated transcript 1 (PCAT-1) in CRC (71, 72). In addition, SNPs in lncRNA promoter region also modulate the expression and function of the lncRNA. Intriguingly, Li et al. found that the presence of a SNP rs67085638 in the 3′ UTR of CCAT 1 increases the expression of CCAT 1 (73). Other than SNPs, genomic rearrangements such as deletions, amplifications, or translocations within lncRNA loci may also alter its expression (74). However, there are currently no studies focusing on this aspect.

**FIGURE 2:**
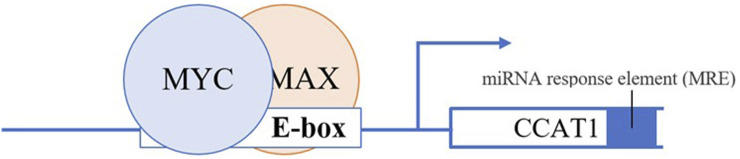
c-Myc dimerizes with a protein called Max to form a transcriptional competent factor complex, this complex then binds the enhancer box (E-box) of CCAT1 at the promoter region thereby augmenting the expression of CCAT1.

Dysregulation of CCAT 1 also mediated by transcriptional regulation of key transcription factor c-MYC (44). CCAT 1 and c-MYC seem to form a double-positive feedback loop to enhance the expression of each other. Growing evidence indicates that the transcription of CCAT 1 can be activated and upregulated by c-MYC (44). Overexpression of c-MYC reciprocally augments the expression of CCAT 1 by binding to the E-box in the promoter region of CCAT 1, consequently accelerating the development and metastasis progress of CRC, and *vice versa* (Figure 2) (44). Interestingly, oncogenic SNP rs6983267 within the *c-MYC* enhancer region increase CCAT 1 expression by long-range interaction with CCAT 1 promoter region (45). In addition, studies suggest that overexpression of CAMP responsive element binding protein 1 (CREB1), a phosphorylation-dependent transcription factor cause upregulation of CCAT1 (75).

Besides genetic alterations, epigenetic regulation such as DNA methylation, gene imprinting, chromatin remodeling, histone modification as well as non-coding RNAs regulation cause lncRNAs dysregulation (76). Studies demonstrate that the chromatin state of lncRNAs has been modified during diseases and this affects the expression of lncRNAs (77). For instance, the transcription of lncRNA maternally expressed gene 3 (MEG3) is repressed notably in hepatocellular cancer due to hypermethylation in its promoter region (77).

## Biological Functions of CCAT 1 in CRC

Many studies reveal that CCAT 1 promotes tumorigenesis in CRC by different mechanisms of action. As stated above, knockdown of CCAT 1 reduces colon cancer cell proliferation, reverses CRC cell invasion and metastasis, and improves CRC cell apoptosis (28, 45, 47). All these results indicate that CCAT 1 does exhibit oncogenic activities in CRC.

Thus, how CCAT 1 facilitates tumorigenesis in CRC? Current studies suggest that CCAT 1 induces CRC cell proliferation through upregulating oncoproteins c-MYC and oncogenic mRNA tumor suppressor candidate 3 (TUSC3), the target of miR-181b-5p in CRC cells (24, 49); enhances glucose metabolism to provide energy supply for the growth of colon cancer cells (78); facilitates CRC cell migration and invasion through accelerating EMT process and negatively modulate miR-218 as well as hsa-miR-4679 (67, 69), and lastly inhibits colon cancer cell apoptosis by sponging miR-181a-5p (Table 2) (47).

**TABLE 2:** Biological functions of CCAT1 in CRC.

Function	CCAT1 promotes CRC cell proliferation	CCAT1 facilitates CRC cell migration and invasion	CCAT1 inhibits CRC cell apoptosis
Mechanism of action	CCAT1 upregulates glycolytic pathway in colon cancer cells	CCAT1 sponges hsa-miR-4679 thereby upregulating GNG10	CCAT1 sponges miR-181a-5p thereby downregulating proapoptotic protein BAX
	CCAT1 sponges miR-181b-5p thereby upregulating Tumor Suppressor Candidate 3 (TUSC3)	CCAT1 sponges miR-218 thereby upregulating vascular endothelial growth factor (VEGF)	
	CCAT1 upregulates c-MYC transcription factor	CCAT1 accelerates EMT process	

### CCAT 1 Promotes CRC Cell Proliferation

CCAT1-L promotes CRC cell proliferation *via* upregulation of c-MYC transcription factor, which has been discussed previously (24). Furthermore, CCAT 1 also promotes CRC cell proliferation through an axis called CCAT1/miR-181b-5p/TUSC3 (49). By sponging miR-181b-5p in CRC cells, CCAT 1 positively regulates the expression of TUSC3 which in turn promote proliferation, migration, invasion, and accelerates tumor growth (49). CCAT 1 upregulates the glycolytic pathway in colon cancer cells by increasing the expression levels of glycolysis rate-limiting enzymes in colon cancer cells and promoting lactic acid production (78). This action provides energy supply for the proliferation of colon cancer cells as malignant tumors primarily rely on the glycolytic pathway for energy supply (78). Cui et al. also show that high glucose levels or hyperglycemia enhance the oncogenic effect of CCAT 1 on colon cancer cell proliferation, anti-apoptotic and migration (78).

### CCAT 1 Facilitates CRC Cell Migration and Invasion

Evidence reveals that CCAT 1 facilitates the migration and invasion of colon cancer cells by accelerating epithelial-mesenchymal transition (EMT) process, in which the tight junctions between epithelial cells undergo dissolution, the polarity between apical and basal domains of epithelial cells are disrupted, and the cytoskeletal reorganized abnormally (79, 80). EMT process enables cancer cells to achieve invasive and migrative abilities so that they can isolate from primary tumor to invade and metastasize to distant organs such as the liver (79, 80). In CRC, CCAT 1 expression is associated with the expression of EMT markers, which are N-cadherin and E-cadherin (79). N-cadherin is a mesenchymal marker of the EMT while E-cadherin is an epithelial marker expressed in most normal epithelial tissues (81). When CCAT 1 is overexpressed, the expression of E-cadherin is downregulated whereas the expression of critical indicators of EMT including N-cadherin and vimentin is upregulated (82). This suggests that CCAT 1 may mediate CRC cell migration and invasion by accelerating EMT process.

Besides, overexpression of CCAT 1 can increase vascular endothelial growth factor (VEGF) expression by sponging miR-218, leading to an increase in CRC cell viability, proliferation, migration and invasion (67). MiR-218 has been suggested to inhibit the expression of VEGF that promotes angiogenesis, which is essential for cancer development and growth (67, 83). The study demonstrates that CCAT 1 and miR-218 have complementary binding sites. CCAT 1 can directly bind to miR-218 to inhibit its suppressive role on VEGF, thereby promoting CRC cell migration, invasion and viability (67).

A recent study demonstrates that CCAT 1 promotes progression of CRC *via* interaction between hsa-miR-4679 and GNG10 (69). GNG10 is a subunit of G-protein and was previously shown to have a potential role in melanoma tumorigenesis (84). In CRC, GNG10 was highly expressed whereas hsa-miR-4679 has low expression (69). By sponging tumor suppressor hsa-miR-4679, CCAT 1 upregulates GNG10 expression leading to CRC cell migration and invasion.

### CCAT 1 Inhibits CRC Cell Apoptosis

CCAT 1 acts as competing endogenous RNAs (ceRNAs) to regulate tumor suppressors and apoptosis signaling pathways in CRC. For instance, CCAT 1 serves as a miRNA sponge for tumor suppressive miR-181a-5p that regulates the expression of apoptosis-related proteins BAX and B-cell lymphoma-2 (BCL-2) which are involved in the p53 signaling pathway and subsequently affect the proliferation of CRC cells (47). As CCAT 1 contains MRE that captures miR-181a-5p, it plays a tumor promoter role by binding to miR-181a-5p and abating the effect of miR-181a-5p on its own target pro-apoptotic protein BAX in CRC cells (47). As such, the expression level of BAX proteins reduces, leading to a reduction in CRC cell apoptosis (47). In opposite, the downregulation of CCAT 1 or upregulation of miR-181a-5p increases the expression levels of BAX *via* the p53 signaling pathway, resulting in accelerated CRC cell apoptosis (47).

## Roles of CCAT 1 in Resistance to Chemotherapy

Besides surgery, traditional chemotherapy drugs and advanced molecular target therapy are important means to destroy cancer cells, they can be used to manage patients with primary and metastatic CRC (85). However, the treatment of CRC has been challenging because it is a molecularly heterogeneous disease in which the tumors harbor distinct molecular features with different levels of sensitivity to treatments (86). Moreover, all malignant colon cells manifest chemotherapy-related resistance (87). Therefore, in some cases, chemotherapy alone can hardly provide a complete cure, and this brings up a critical problem (87).

5-fluorouracil (5-FU) is the first-line chemotherapy for CRC patients. It is a synthetic fluorinated pyrimidine analogue that can interfere with DNA synthesis by irreversibly inhibiting the action of thymidylate synthase or incorporating its metabolites into DNA, thus leading to DNA damage and cell death (88). However, it is found that CRC patients often develop resistance to 5-FU-based chemotherapies, and this leads to a poor prognosis for patients. A recent study shows that nearly half of the patients that have metastatic CRC are resistant to 5-FU-based chemotherapies, therefore, finding out the resistance mechanisms is of upmost important (22). Chun Yang et al. demonstrate that downregulation of CCAT 1 significantly reverses the drug resistance of 5-FU-resistant colonic neoplasm cell lines by accelerating cell apoptosis (22). Although the underlying mechanism remains unclear, it provides a new direction for colon cancer treatment.

Another strong chemotherapy drug used to treat CRC patients is paclitaxel (PTX) (89). PTX exerts anti-tumor functions by inhibiting CRC cell proliferation through cell cycle arrest and preventing angiogenic features of endothelial cells (89). Fascin Actin-Bundling Protein 1 (FSCN1), which is the functional target of miR-24-3p, plays a key role in miR-24-3p-mediated sensitivity to paclitaxel (90). FSCN1 is an actin binding protein, it promotes cell migration, adhesion, invasion through EMT process, and its expression reduces the chemosensitivity of cancer cells to paclitaxel (91). CCAT 1 enhances chemoresistance of CRC cancer cells to PTX by regulating the expression of miR-24-3p as well as the expression of FSCN1 (90). CCAT 1 negatively modulates the expression of miR-24-3p to elevate the expression of FSCN 1 mRNA, leading to increased chemoresistance of CRC cells to PTX. Interestingly, the presence of the SNP rs67085638 in the CCAT 1 3′ UTR region increases the expression of CCAT 1 and enhances the chemoresistance to PTX in CRC cells (90). Nonetheless, downregulation of CCAT 1 can significantly restore the sensitivity of colon cancer cells to PTX (90). This highlights that CCAT 1 may be the hope in future therapeutic approaches in CRC.

## Conclusion

CCAT 1 is a pivotal oncogenic lncRNA that may serve as a potential biomarker in the screening, diagnosis, prognosis, and treatment of CRC. All in all, the development of reliable diagnostic assays and effective therapeutic methods will be facilitated by a better knowledge of the roles of CCAT1 in CRC including its interaction with miRNAs, and this could significantly improve the long-term survival rate of CRC patients and reduce CRC morbidity and mortality. However, even though several studies have looked at the mechanisms of action of CCAT 1 in CRC, the factors that cause dysregulation of CCAT 1 in CRC are still not well understood. Moreover, there has been very little discussion about the underlying mechanism that causes CCAT 1 dysregulation in CRC, particularly epigenetic regulation. Other than that, many functional targets still have not been proven to correlate with CCAT 1 expression in colorectal cancer. Therefore it is likely to be some time before CCAT 1 can be clinically used as a biomarker in CRC. Further exploration is required to allow gaps in our current knowledge to be filled and for research in this area to progress further.

## References

[1] Ferlay J Ervik M Lam F Colombet M Mery L Piñeros M Global Cancer Observatory: Cancer Today. Lyon, France (2020). Available from: https://gco.iarc.fr/today (accessed Jun 30, 2022).

[2] Cross W Kovac M Mustonen V Temko D Davis H Baker AM The Evolutionary Landscape of Colorectal Tumorigenesis. Nat Ecol Evol (2018) 2(10):1661–72. 10.1038/s41559-018-0642-z 30177804 PMC6152905

[3] Hong Q Li B Cai X Lv Z Cai S Zhong Y Transcriptomic Analyses of the Adenoma-Carcinoma Sequence Identify Hallmarks Associated with the Onset of Colorectal Cancer. Front Oncol (2021) 11:704531. 10.3389/fonc.2021.704531 34458146 PMC8387103

[4] Schliemann D Paramasivam D Dahlui M Cardwell CR Somasundaram S Ibrahim Tamin NSB Change in Public Awareness of Colorectal Cancer Symptoms Following the Be Cancer Alert Campaign in the Multi-Ethnic Population of Malaysia. BMC Cancer (2020) 20(1):252–12. 10.1186/s12885-020-06742-3 32213173 PMC7093961

[5] Duineveld LAM van Asselt KM Bemelman WA Smits AB Tanis PJ van Weert HCPM Symptomatic and Asymptomatic Colon Cancer Recurrence: A Multicenter Cohort Study. Ann Fam Med (2016) 14(3):215–20. 10.1370/afm.1919 27184991 PMC4868559

[6] Kuipers EJ Rösch T Bretthauer M. Colorectal Cancer Screening—Optimizing Current Strategies and New Directions. Nat Rev Clin Oncol (2013) 10(3):130–42. 10.1038/nrclinonc.2013.12 23381005

[7] Kalmár A Nagy ZB Galamb O Csabai I Bodor A Wichmann B Genome-wide Expression Profiling in Colorectal Cancer Focusing on lncRNAs in the Adenoma-Carcinoma Transition. BMC Cancer (2019) 19(1):1059. 10.1186/s12885-019-6180-5 31694571 PMC6836529

[8] Dahariya S Paddibhatla I Kumar S Raghuwanshi S Pallepati A Gutti RK. Long Non-coding RNA: Classification, Biogenesis and Functions in Blood Cells. Mol Immunol (2019) 112:82–92. 10.1016/j.molimm.2019.04.011 31079005

[9] Carlevaro-Fita J Lanzós A Feuerbach L Hong C Mas-Ponte D Pedersen JS Cancer LncRNA Census Reveals Evidence for Deep Functional Conservation of Long Noncoding RNAs in Tumorigenesis. Commun Biol (2020) 3(1):56. 10.1038/s42003-019-0741-7 32024996 PMC7002399

[10] Schmitt AM Chang HY. Long Noncoding RNAs in Cancer Pathways. Cancer Cell (2016) 29(4):452–63. 10.1016/J.CCELL.2016.03.010 27070700 PMC4831138

[11] Xie X Tang B Xiao Y-F Xie R Li B-S Dong H Long Non-coding RNAs in Colorectal Cancer. Oncotarget (2016) 7(5):5226–39. 10.18632/oncotarget.6446 26637808 PMC4868682

[12] Nissan A Stojadinovic A Mitrani-Rosenbaum S Halle D Grinbaum R Roistacher M Colon Cancer Associated Transcript-1: A Novel RNA Expressed in Malignant and Pre-malignant Human Tissues. Int J Cancer (2012) 130(7):1598–606. 10.1002/ijc.26170 21547902

[13] Mizrahi I Mazeh H Grinbaum R Beglaibter N Wilschanski M Pavlov V Colon Cancer Associated Transcript-1 (CCAT1) Expression in Adenocarcinoma of the Stomach. J Cancer (2015) 6(2):105–10. 10.7150/jca.10568 25561974 PMC4280392

[14] Luo J Tang L Zhang J Ni J Zhang H Zhang L Long Non-coding RNA CARLo-5 Is a Negative Prognostic Factor and Exhibits Tumor Pro-oncogenic Activity in Non-small Cell Lung Cancer. Tumor Biol (2014) 35(11):11541–9. 10.1007/s13277-014-2442-7 25129441

[15] Zhang X-F Liu T Li Y Li S. Overexpression of Long Non-coding RNA CCAT1 Is a Novel Biomarker of Poor Prognosis in Patients with Breast Cancer. Int J Clin Exp Pathol (2015) 8(8):9440–5. 26464701 PMC4583933

[16] Cao Y Shi H Ren F Jia Y Zhang R. Long Non-coding RNA CCAT1 Promotes Metastasis and Poor Prognosis in Epithelial Ovarian Cancer. Exp Cel Res (2017) 359(1):185–94. 10.1016/j.yexcr.2017.07.030 28754469

[17] Ma M-Z Chu B-F Zhang Y Weng M-Z Qin Y-Y Gong W Long Non-coding RNA CCAT1 Promotes Gallbladder Cancer Development via Negative Modulation of miRNA-218-5p. Cell Death Dis (2015) 6:e1583. 10.1038/cddis.2014.541 25569100 PMC4669740

[18] Deng L Yang SB Xu FF Zhang JH. Long Noncoding RNA CCAT1 Promotes Hepatocellular Carcinoma Progression by Functioning as Let-7 Sponge. J Exp Clin Cancer Res (2015) 34(1):18–0. 10.1186/s13046-015-0136-7 25884472 PMC4339002

[19] You Z Liu C Wang C Ling Z Wang Y Wang Y LncRNA CCAT1 Promotes Prostate Cancer Cell Proliferation by Interacting with DDX5 and MIR-28-5P. Mol Cancer Ther (2019) 18(12):2469–79. 10.1158/1535-7163.MCT-19-0095 31387890

[20] Saleh E Mohamed M El-Khazragy N Abdelmaksoud R. Assessment of the Lnc-CCAT1/miR-155a Regulatory Network in Acute Myeloid Leukemia. Clin Oncol Res (2019) 2019(4):1–8. 10.31487/J.COR.2019.04.04

[21] Ma D Cao Y Wang Z He J Chen H Xiong H CCAT1 lncRNA Promotes Inflammatory Bowel Disease Malignancy by Destroying Intestinal Barrier via Downregulating miR-185-3p. Inflamm Bowel Dis (2019) 25(5):862–74. 10.1093/ibd/izy381 30615124

[22] Yang C Pan Y Deng SP. Downregulation of lncRNA CCAT1 Enhances 5-fluorouracil Sensitivity in Human colon Cancer Cells. BMC Mol Cel Biol (2019) 20(1):9. 10.1186/s12860-019-0188-1 PMC6480879 31039730

[23] Liu Z Chen Q Hann SS. The Functions and Oncogenic Roles of CCAT1 in Human Cancer. Biomed Pharmacother (2019) 115:108943. 10.1016/j.biopha.2019.108943 31078038

[24] Xiang J-F Yin Q-F Chen T Zhang Y Zhang X-O Wu Z Human Colorectal Cancer-specific CCAT1-L lncRNA Regulates Long-Range Chromatin Interactions at the MYC Locus. Cell Res (2014) 24(5):513–31. 10.1038/cr.2014.35 24662484 PMC4011346

[25] Yu Q Zhou X Xia Q Shen J Yan J Zhu J Long Non-coding RNA CCAT1 that Can Be Activated by C-Myc Promotes Pancreatic Cancer Cell Proliferation and Migration. Am J Transl Res (2016) 8(12):5444–54. 28078015 PMC5209495

[26] Liu Z Ma C Tang X Tang Q Lou L Yu Y The Reciprocal Interaction between LncRNA CCAT1 and miR-375-3p Contribute to the Downregulation of IRF5 Gene Expression by Solasonine in HepG2 Human Hepatocellular Carcinoma Cells. Front Oncol (2019) 9:1081. 10.3389/fonc.2019.01081 31681610 PMC6813207

[27] Chen L Wang W Cao L Li Z Wang X. Long Non-coding RNA CCAT1 Acts as a Competing Endogenous RNA to Regulate Cell Growth and Differentiation in Acute Myeloid Leukemia. Mol Cell (2016) 39(4):330–6. 10.14348/molcells.2016.2308 PMC4844940 26923190

[28] Chen Y Xie H Gao Q Zhan H Xiao H Zou Y Colon Cancer Associated Transcripts in Human Cancers. Biomed Pharmacother (2017) 94:531–40. 10.1016/j.biopha.2017.07.073 28779711

[29] Fang H Liu H-M Wu W-H Liu H Pan Y Li W-J. Upregulation of Long Noncoding RNA CCAT1-L Promotes Epithelial-Mesenchymal Transition in Gastric Adenocarcinoma. Onco Targets Ther (2018) 11:5647–55. 10.2147/OTT.S170553 30254457 PMC6141104

[30] Zhang L Zhu C Wang L Li H. KNOCKDOWN OF LONG NON-CODING RNA CCAT1-L CAN INHIBIT PROLIFERATION AND METASTASIS OF HEPATOMA CELLS. Acta Med Mediterranea (2019) 35:1667. 10.19193/0393-6384_2019_3_261

[31] Alaiyan B Ilyayev N Stojadinovic A Izadjoo M Roistacher M Pavlov V Differential Expression of colon Cancer Associated Transcript1 (CCAT1) along the Colonic Adenoma-Carcinoma Sequence. BMC Cancer (2013) 13:196. 10.1186/1471-2407-13-196 23594791 PMC3639026

[32] Barbagallo C Brex D Caponnetto A Cirnigliaro M Scalia M Magnano A LncRNA UCA1, Upregulated in CRC Biopsies and Downregulated in Serum Exosomes, Controls mRNA Expression by RNA-RNA Interactions. Mol Ther Nucleic Acids (2018) 12:229–41. 10.1016/j.omtn.2018.05.009 30195762 PMC6023947

[33] Thean LF Blöcker C Li HH Lo M Wong M Tang CL Enhancer-derived Long Non-coding RNAs CCAT1 and CCAT2 at Rs6983267 Has Limited Predictability for Early Stage Colorectal Carcinoma Metastasis. Sci Rep (2021) 11(1):404. 10.1038/s41598-020-79906-7 33432117 PMC7801656

[34] Siddique H Al-Ghafari A Choudhry H AlTurki S Alshaibi H al Doghaither H Long Noncoding RNAs as Prognostic Markers for Colorectal Cancer in Saudi Patients. Genet Test Mol Biomarkers (2019) 23(8):509–14. 10.1089/gtmb.2018.0308 31328973

[35] Gharib E Nazemalhosseini‐Mojarad E Baghdar K Nayeri Z Sadeghi H Rezasoltani S Identification of a Stool Long Non‐coding RNAs Panel as a Potential Biomarker for Early Detection of Colorectal Cancer. J Clin Lab Anal (2021) 35(2):e23601. 10.1002/jcla.23601 33094859 PMC7891513

[36] Zhao W Song M Zhang J Kuerban M Wang H. Combined Identification of Long Non-coding RNA CCAT1 and HOTAIR in Serum as an Effective Screening for Colorectal Carcinoma. Int J Clin Exp Pathol (2015) 8(11):14131–40. 26823726 PMC4713512

[37] Kam Y Rubinstein A Naik S Djavsarov I Halle D Ariel I Detection of a Long Non-coding RNA (CCAT1) in Living Cells and Human Adenocarcinoma of colon Tissues Using FIT-PNA Molecular Beacons. Cancer Lett (2014) 352(1):90–6. 10.1016/j.canlet.2013.02.014 23416875

[38] Oldenhuis CNAM Oosting SF Gietema JA de Vries EGE. Prognostic versus Predictive Value of Biomarkers in Oncology. Eur J Cancer (2008) 44(7):946–53. 10.1016/j.ejca.2008.03.006 18396036

[39] Shi D Wu F Gao F Qing X Shao Z. Prognostic Value of Long Non-coding RNA CCAT1 Expression in Patients with Cancer: A Meta-Analysis. PLoS One (2017) 12(6):e0179346. 10.1371/journal.pone.0179346 28594897 PMC5464649

[40] Ozawa T Matsuyama T Toiyama Y Takahashi N Ishikawa T Uetake H CCAT1 and CCAT2 Long Noncoding RNAs, Located within the 8q.24.21 ‘gene Desert’, Serve as Important Prognostic Biomarkers in Colorectal Cancer. Ann Oncol (2017) 28(8):1882–8. 10.1093/annonc/mdx248 28838211 PMC5834045

[41] Zhang Z Xie H Liang D Huang L Liang F Qi Q Long Non-coding RNA CCAT1 as a Diagnostic and Prognostic Molecular Marker in Various Cancers: a Meta-Analysis. Oncotarget (2018) 9(34):23695–703. 10.18632/oncotarget.24923 29805767 PMC5955114

[42] Alqahtani A Choucair K Ashraf M Hammouda DM Alloghbi A Khan T Bromodomain and Extra-terminal Motif Inhibitors: a Review of Preclinical and Clinical Advances in Cancer Therapy. Future Sci OA (2019) 5(3):FSO372. 10.4155/fsoa-2018-0115 30906568 PMC6426170

[43] McCleland ML Mesh K Lorenzana E Chopra VS Segal E Watanabe C CCAT1 Is an Enhancer-Templated RNA that Predicts BET Sensitivity in Colorectal Cancer. J Clin Invest (2016) 126(2):639–52. 10.1172/JCI83265 26752646 PMC4731162

[44] He X Tan X Wang X Jin H Liu L Ma L C-Myc-activated Long Noncoding RNA CCAT1 Promotes colon Cancer Cell Proliferation and Invasion. Tumour Biol (2014) 35(12):12181–8. 10.1007/s13277-014-2526-4 25185650

[45] Kim T Cui R Jeon Y-J Lee J-H Lee JH Sim H Long-range Interaction and Correlation between MYC Enhancer and Oncogenic Long Noncoding RNA CARLo-5. Proc Natl Acad Sci U S A (2014) 111(11):4173–8. 10.1073/pnas.1400350111 24594601 PMC3964128

[46] Li J Qi Y. Ginsenoside Rg3 Inhibits Cell Growth, Migration and Invasion in Caco-2 Cells by Downregulation of lncRNA CCAT1. Exp Mol Pathol (2019) 106:131–8. 10.1016/j.yexmp.2019.01.003 30633886

[47] Shang A Wang W Gu C Chen W Lu W Sun Z Long Non-coding RNA CCAT1 Promotes Colorectal Cancer Progression by Regulating miR-181a-5p Expression. Aging (2020) 12(9):8301–20. 10.18632/aging.103139 32380476 PMC7244037

[48] Sanchez Calle A Kawamura Y Yamamoto Y Takeshita F Ochiya T. Emerging Roles of Long Non-coding RNA in Cancer. Cancer Sci (2018) 109(7):2093–100. 10.1111/cas.13642 29774630 PMC6029823

[49] Chen S Liu Y Wang Y Xue Z. LncRNA CCAT1 Promotes Colorectal Cancer Tumorigenesis via A miR-181b-5p/TUSC3 Axis. Onco Targets Ther (2019) 12:9215–25. 10.2147/OTT.S216718 31807005 PMC6842281

[50] Grisanzio C Freedman ML. Chromosome 8q24-Associated Cancers and MYC. Genes Cancer (2010) 1(6):555–9. 10.1177/1947601910381380 21779458 PMC3092220

[51] Cole MD. MYC Association with Cancer Risk and a New Model of MYC-Mediated Repression. Cold Spring Harb Perspect Med (2014) 4(7):a014316. 10.1101/cshperspect.a014316 24985129 PMC4066640

[52] Huppi K Pitt JJ Wahlberg BM Caplen NJ. The 8q24 Gene Desert: an Oasis of Non-coding Transcriptional Activity. Front Genet (2012) 3:69. 10.3389/fgene.2012.00069 22558003 PMC3339310

[53] Lee KS Kwak Y Nam KH Kim D-W Kang S-B Choe G c-MYC Copy-Number Gain Is an Independent Prognostic Factor in Patients with Colorectal Cancer. PLoS One (2015) 10(10):e0139727. 10.1371/journal.pone.0139727 26426996 PMC4591346

[54] Dang Cv. MYC on the Path to Cancer. Cell (2012) 149(1):22–35. 10.1016/j.cell.2012.03.003 22464321 PMC3345192

[55] Iaccarino I. lncRNAs and MYC: An Intricate Relationship. Int J Mol Sci (2017) 18(7):1497. 10.3390/ijms18071497 28704924 PMC5535987

[56] Pomerantz MM Ahmadiyeh N Jia L Herman P Verzi MP Doddapaneni H The 8q24 Cancer Risk Variant Rs6983267 Shows Long-Range Interaction with MYC in Colorectal Cancer. Nat Genet (2009) 41(8):882–4. 10.1038/ng.403 19561607 PMC2763485

[57] Zanke BW Greenwood CM Rangrej J Kustra R Tenesa A Farrington SM Genome-wide Association Scan Identifies a Colorectal Cancer Susceptibility Locus on Chromosome 8q24. Nat Genet (2007) 39(8):989–94. 10.1038/ng2089 17618283

[58] Danielsen SA Eide PW Nesbakken A Guren T Leithe E Lothe RA. Portrait of the PI3K/AKT Pathway in Colorectal Cancer. Biochim Biophys Acta (2015) 1855(1):104–21. 10.1016/j.bbcan.2014.09.008 25450577

[59] Zhu J Blenis J Yuan J. Activation of PI3K/Akt and MAPK Pathways Regulates Myc-Mediated Transcription by Phosphorylating and Promoting the Degradation of Mad1. Proc Natl Acad Sci U S A (2008) 105(18):6584–9. 10.1073/pnas.0802785105 18451027 PMC2373325

[60] Elbadawy M Usui T Yamawaki H Sasaki K. Emerging Roles of C-Myc in Cancer Stem Cell-Related Signaling and Resistance to Cancer Chemotherapy: A Potential Therapeutic Target against Colorectal Cancer. Int J Mol Sci (2019) 20(9):2340. 10.3390/ijms20092340 31083525 PMC6539579

[61] White NM Maher CA. The Potential Use of lncRNAs Found in the 8q24 Region as Biomarkers for colon Cancer. Ann Oncol (2017) 28(8):1688–9. 10.1093/annonc/mdx337 28838213

[62] de Falco G Ambrosio MR Fuligni F Onnis A Bellan C Rocca BJ Burkitt Lymphoma beyond MYC Translocation: N-MYC and DNA Methyltransferases Dysregulation. BMC Cancer (2015) 15:668. 10.1186/s12885-015-1661-7 26453442 PMC4600215

[63] Kadauke S Blobel GA. Chromatin Loops in Gene Regulation. Biochim Biophys Acta (2009) 1789(1):17–25. 10.1016/j.bbagrm.2008.07.002 18675948 PMC2638769

[64] Gmerek L Martyniak K Horbacka K Krokowicz P Scierski W Golusinski P MicroRNA Regulation in Colorectal Cancer Tissue and Serum. PLoS One (2019) 14(8):e0222013. 10.1371/journal.pone.0222013 31469874 PMC6716664

[65] Wu M Li W Huang F Sun J Li K Shi J Comprehensive Analysis of the Expression Profiles of Long Non-coding RNAs with Associated ceRNA Network Involved in the Colon Cancer Staging and Progression. Scientific Rep (2019) 9(1):1–10. 10.1038/s41598-019-52883-2 PMC6858342 31729423

[66] Schetter AJ Okayama H Harris CC. The Role of microRNAs in Colorectal Cancer. Cancer J (2012) 18(3):244–52. 10.1097/PPO.0b013e318258b78f 22647361 PMC3397427

[67] Gu C Zou S He C Zhou J Qu R Wang Q Long Non-coding RNA CCAT1 Promotes Colorectal Cancer Cell Migration, Invasiveness and Viability by Upregulating VEGF via Negative Modulation of microRNA-218. Exp Ther Med (2020) 19(4):2543–50. 10.3892/etm.2020.8518 32256733 PMC7086191

[68] Li B Shi C Zhao J Li B. Long Noncoding RNA CCAT1 Functions as a ceRNA to Antagonize the Effect of miR-410 on the Down-Regulation of ITPKB in Human HCT-116 and HCT-8 Cells. Oncotarget (2017) 8(54):92855–63. 10.18632/oncotarget.21612 29190961 PMC5696227

[69] Wang N Li J He J Jing Y-G Zhao W-D Yu W-J Knockdown of lncRNA CCAT1 Inhibits the Progression of Colorectal Cancer via Hsa-miR-4679 Mediating the Downregulation of GNG10. J Immunol Res (2021) 2021:8930813. 10.1155/2021/8930813 35005034 PMC8739552

[70] Wang W He Y Rui J Xu M-Q. miR-410 Acts as an Oncogene in Colorectal Cancer Cells by Targeting Dickkopf-Related Protein 1 via the Wnt/β-Catenin Signaling Pathway. Oncol Lett (2019) 17(1):807–14. 10.3892/ol.2018.9710 30655833 PMC6313057

[71] Ling H Spizzo R Atlasi Y Nicoloso M Shimizu M Redis RS CCAT2, a Novel Noncoding RNA Mapping to 8q24, Underlies Metastatic Progression and Chromosomal Instability in colon Cancer. Genome Res (2013) 23(9):1446–61. 10.1101/gr.152942.112 23796952 PMC3759721

[72] Yang M-L Huang Z Wu L-N Wu R Ding H-X Wang B-G. lncRNA-PCAT1 Rs2632159 Polymorphism Could Be a Biomarker for Colorectal Cancer Susceptibility. Biosci Rep (2019) 39(7). 10.1042/BSR20190708 PMC6629943 31253700

[73] Li Y Jing F Ding Y He Q Zhong Y Fan C. Long Noncoding RNA CCAT1 Polymorphisms Are Associated with the Risk of Colorectal Cancer. Cancer Genet (2018) 222–223:13–9. 10.1016/j.cancergen.2018.02.003 29666003

[74] Aznaourova M Schmerer N Schmeck B Schulte LN. Disease-Causing Mutations and Rearrangements in Long Non-coding RNA Gene Loci. Front Genet (2020) 11:527484. 10.3389/fgene.2020.527484 33329688 PMC7735109

[75] Li B Zheng L Ye J Zhang C Zhou J Huang Q CREB1 Contributes Colorectal Cancer Cell Plasticity by Regulating lncRNA CCAT1 and NF-κB Pathways. Sci China Life Sci (2022) 65(8):1481–97. 10.1007/s11427-022-2108-x 35696016

[76] Guo X Hua Y. CCAT1: an Oncogenic Long Noncoding RNA in Human Cancers. J Cancer Res Clin Oncol (2017) 143(4):555–62. 10.1007/s00432-016-2268-3 27638771 PMC11819113

[77] Wu Z Liu X Liu L Deng H Zhang J Xu Q Regulation of lncRNA Expression. Cell Mol Biol Lett (2014) 19(4):561–75. 10.2478/s11658-014-0212-6 25311814 PMC6275606

[78] Cui G Huang Y Feng W Yao Y Zhou H Li X Colon Cancer-Associated Transcript-1 Enhances Glucose Metabolism and colon Cancer Cell Activity in a High-Glucose Environment *In Vitro* and *In Vivo* . J Gastrointest Oncol (2020) 11(6):1164–85. 10.21037/jgo-20-474 33456991 PMC7807285

[79] Ye Z Zhou M Tian B Wu B Li J. Expression of lncRNA-CCAT1, E-Cadherin and N-Cadherin in Colorectal Cancer and its Clinical Significance. Int J Clin Exp Med (2015) 8(3):3707–15. 26064266 PMC4443100

[80] Vu T Datta PK. Regulation of EMT in Colorectal Cancer: A Culprit in Metastasis. Cancers (Basel) (2017) 9(12):171. 10.3390/cancers9120171 29258163 PMC5742819

[81] Yan X Yan L Liu S Shan Z Tian Y Jin Z. N-Cadherin, a Novel Prognostic Biomarker, Drives Malignant Progression of Colorectal Cancer. Mol Med Rep (2015) 12(2):2999–3006. 10.3892/mmr.2015.3687 25936636

[82] Lin H Cheng W Yan H Zhang X. Overexpression of the Long Noncoding RNA CCAT1 Promotes Metastasis via Epithelial-To-Mesenchymal Transition in Lung Adenocarcinoma. Oncol Lett (2018) 16(2):1809–14. 10.3892/ol.2018.8813 30008869 PMC6036492

[83] Carmeliet P. VEGF as a Key Mediator of Angiogenesis in Cancer. Oncology (2005) 69:4–10. 10.1159/000088478 16301830

[84] Cárdenas-Navia LI Cruz P Lin JC Rosenberg SA Samuels Y. NISC Comparative Sequencing Program. Novel Somatic Mutations in Heterotrimeric G Proteins in Melanoma. Cancer Biol Ther (2010) 10(1):33–7. 10.4161/cbt.10.1.11949 20424519 PMC3040832

[85] Xie Y-H Chen Y-X Fang J-Y. Comprehensive Review of Targeted Therapy for Colorectal Cancer. Signal Transduct Target Ther (2020) 5(1):22. 10.1038/s41392-020-0116-z 32296018 PMC7082344

[86] Dagogo-Jack I Shaw AT. Tumour Heterogeneity and Resistance to Cancer Therapies. Nat Rev Clin Oncol (2018) 15(2):81–94. 10.1038/nrclinonc.2017.166 29115304

[87] Skarkova V Kralova V Vitovcova B Rudolf E. Selected Aspects of Chemoresistance Mechanisms in Colorectal Carcinoma-A Focus on Epithelial-To-Mesenchymal Transition, Autophagy, and Apoptosis. Cells (2019) 8(3):234. 10.3390/cells8030234 30871055 PMC6468859

[88] van der Jeught K Xu H-C Li Y-J Lu X-B Ji G. Drug Resistance and New Therapies in Colorectal Cancer. World J Gastroenterol (2018) 24(34):3834–48. 10.3748/wjg.v24.i34.3834 30228778 PMC6141340

[89] Lv C Qu H Zhu W Xu K Xu A Jia B Low-Dose Paclitaxel Inhibits Tumor Cell Growth by Regulating Glutaminolysis in Colorectal Carcinoma Cells. Front Pharmacol (2017) 8:244. 10.3389/fphar.2017.00244 28522974 PMC5415623

[90] Xiao Z-S Zhao L Zhang X-N Li H-X Yin Z-H. Effect of Rs67085638 in Long Non-coding RNA (CCAT1) on colon Cancer Chemoresistance to Paclitaxel through Modulating the microRNA-24-3p and FSCN1. J Cel Mol Med (2021) 25(8):3744–53. 10.1111/jcmm.16210 PMC8051717 33709519

[91] Li X Han X Wei P Yang J Sun J. Knockdown of lncRNA CCAT1 Enhances Sensitivity of Paclitaxel in Prostate Cancer via Regulating miR-24-3p and FSCN1. Cancer Biol Ther (2020) 21(5):452–62. 10.1080/15384047.2020.1727700 32089062 PMC7515504

